# Bifidobacteria and Butyrate-Producing Colon Bacteria: Importance and Strategies for Their Stimulation in the Human Gut

**DOI:** 10.3389/fmicb.2016.00979

**Published:** 2016-06-28

**Authors:** Audrey Rivière, Marija Selak, David Lantin, Frédéric Leroy, Luc De Vuyst

**Affiliations:** Research Group of Industrial Microbiology and Food Biotechnology, Faculty of Sciences and Bioengineering Sciences, Vrije Universiteit BrusselBrussels, Belgium

**Keywords:** bifidobacteria, butyrate-producing colon bacteria, cross-feeding, prebiotics, probiotics, arabinoxylan-oligosaccharides, inulin-type fructans

## Abstract

With the increasing amount of evidence linking certain disorders of the human body to a disturbed gut microbiota, there is a growing interest for compounds that positively influence its composition and activity through diet. Besides the consumption of probiotics to stimulate favorable bacterial communities in the human gastrointestinal tract, prebiotics such as inulin-type fructans (ITF) and arabinoxylan-oligosaccharides (AXOS) can be consumed to increase the number of bifidobacteria in the colon. Several functions have been attributed to bifidobacteria, encompassing degradation of non-digestible carbohydrates, protection against pathogens, production of vitamin B, antioxidants, and conjugated linoleic acids, and stimulation of the immune system. During life, the numbers of bifidobacteria decrease from up to 90% of the total colon microbiota in vaginally delivered breast-fed infants to <5% in the colon of adults and they decrease even more in that of elderly as well as in patients with certain disorders such as antibiotic-associated diarrhea, inflammatory bowel disease, irritable bowel syndrome, obesity, allergies, and regressive autism. It has been suggested that the bifidogenic effects of ITF and AXOS are the result of strain-specific yet complementary carbohydrate degradation mechanisms within cooperating bifidobacterial consortia. Except for a bifidogenic effect, ITF and AXOS also have shown to cause a butyrogenic effect in the human colon, i.e., an enhancement of colon butyrate production. Butyrate is an essential metabolite in the human colon, as it is the preferred energy source for the colon epithelial cells, contributes to the maintenance of the gut barrier functions, and has immunomodulatory and anti-inflammatory properties. It has been shown that the butyrogenic effects of ITF and AXOS are the result of cross-feeding interactions between bifidobacteria and butyrate-producing colon bacteria, such as *Faecalibacterium prausnitzii* (clostridial cluster IV) and *Anaerostipes, Eubacterium*, and *Roseburia* species (clostridial cluster XIVa). These kinds of interactions possibly favor the co-existence of bifidobacterial strains with other bifidobacteria and with butyrate-producing colon bacteria in the human colon.

## Introduction

**Figure 1:**
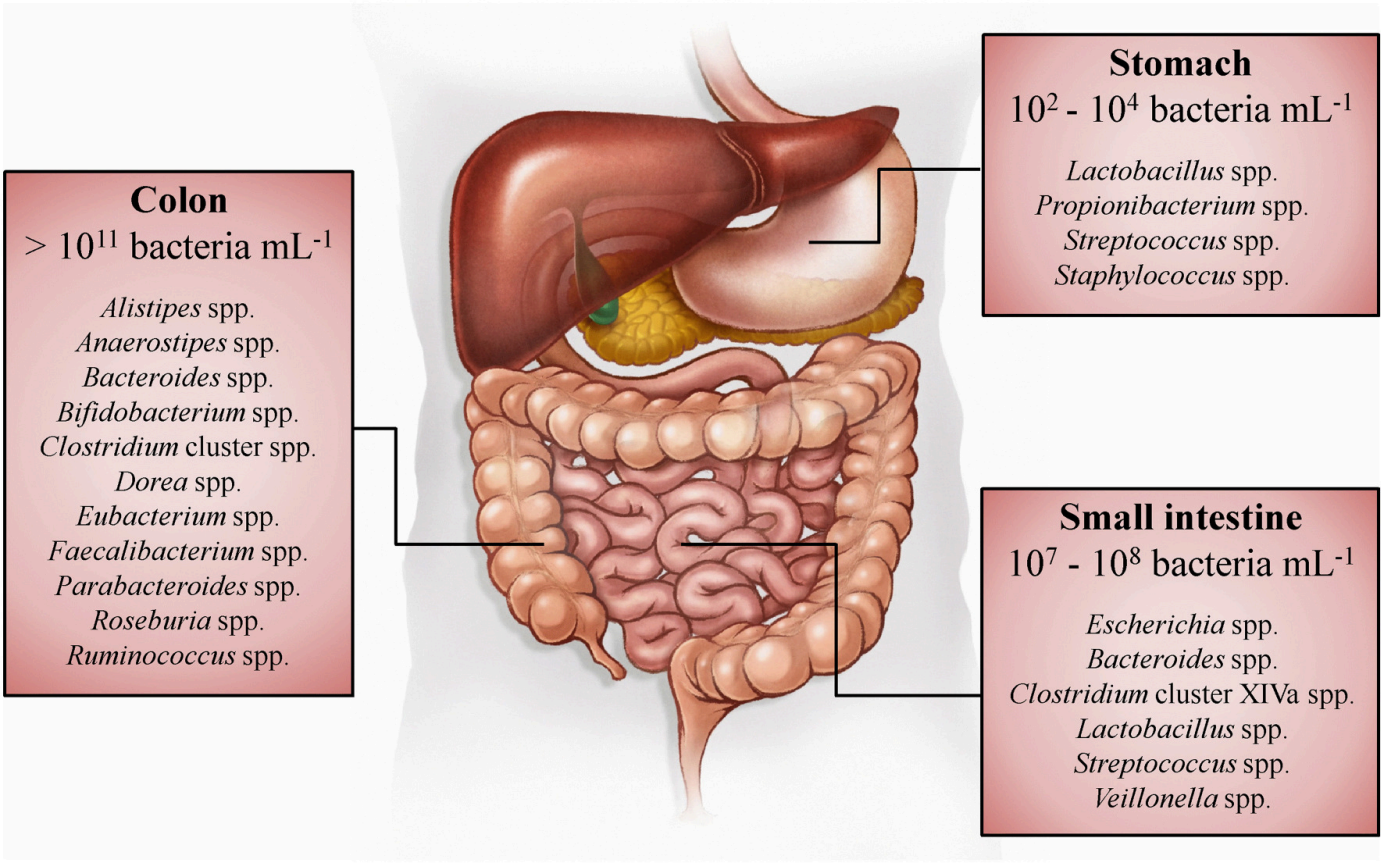
**Spatial distribution and concentrations of bacteria along the gastrointestinal tract of humans (Tuohy and Scott, 2015)**. The dominant genera in the stomach, small intestine, and colon are listed, based on 16S rRNA gene sequence studies (Tap et al., 2009; Zoetendal et al., 2012; Delgado et al., 2013; Walker et al., 2014).

Whereas, the human gut microbiota has been studied in the past mainly in the context of infectious diseases, it is known today that this enormous number of microorganisms has an indispensable role in the normal development and functioning of the human body (O'Hara and Shanahan, 2006; Sommer and Bäckhed, 2013). Within the adult gastrointestinal tract, the colon contains the most dense (>10^11^ bacteria per mL of luminal content) and metabolically active microbiota (Figure 1; Whitman et al., 1998; The Human Microbiome Project Consortium, 2012). The immense number of genes (>100 times the number of genes of the human genome) encoded by this microbiota, expands the host's biochemical and metabolic capabilities substantially (Bäckhed et al., 2005; The Human Microbiome Project Consortium, 2012). Examples of supporting functions of the human gut microbiota are the degradation of otherwise non-digestible food compounds; the transformation of toxic compounds; and the production of essential vitamins, important metabolic end-products, and defending bacteriocins (Sommer and Bäckhed, 2013). Microbial metabolic end-products, which account for one third of the metabolites present in the human blood, play an important role in gut homeostasis and have an impact on host metabolism and health (Wikoff et al., 2009; Hood, 2012; Louis et al., 2014; Sharon et al., 2014; Richards et al., 2016). The short-chain fatty acids (SCFAs) acetate, butyrate, and propionate (typically occurring in a 3:1:1 ratio) are quantitatively (total concentration of 50–150 mM) and metabolically the most important microbial end-products of the human colon fermentation process (Louis et al., 2014), as they display several physiological effects (Table 1).

**Table 1:** **Overview of the physiological effects of the short-chain fatty acids (SCFAs) acetate, propionate, and butyrate produced by human colon bacteria (Hamer et al., 2008; Al-Lahham et al., 2010; Havenaar, 2011; Macfarlane and Macfarlane, 2012; Chang et al., 2014; Louis et al., 2014; Tralongo et al., 2014)**.

**SCFA**	**Physiological effect**
Acetate CH_3_-COO^−^	Reaches the portal vein and is metabolized in various tissues **Intestinal effects** Is a minor energy source for the colon epithelial cells Decreases the pH of the colon (which decreases bile salt solubility, increases mineral absorption, decreases ammonia absorption, and inhibits growth of pathogens) Has anti-inflammatory effects Increases colonic blood flow and oxygen uptake Is used by cross-feeding species as a co-substrate to produce butyrate **Other effects** Is a substrate for cholesterol and fatty acid biosynthesis in the liver Is an energy source for muscle and brain tissue
Propionate CH_3_-CH_2_-COO^−^	Reaches the portal vein and is subsequently taken up by the liver **Intestinal effects** Is a minor energy source for the colon epithelial cells Decreases the pH of the colon (which decreases bile salt solubility, increases mineral absorption, decreases ammonia absorption, and inhibits growth of pathogens) Prevents proliferation and induces apoptosis of colorectal cancer cells Interacts with the immune system Has anti-inflammatory effects **Other effects** Promotes satiety Lowers blood cholesterol levels Decreases liver lipogenesis Improves insulin sensitivity
Butyrate CH_3_-CH_2_-CH_2_-COO^−^	Is mainly taken up by the colon epithelial cells, only small amounts reach the portal vein and the systemic circulation **Intestinal effects** Is the preferred energy source for the colon epithelial cells Decreases the pH of the colon (which decreases bile salt solubility, increases mineral absorption, decreases ammonia absorption, and inhibits growth of pathogens) Stimulates proliferation of normal colon epithelial cells Prevents proliferation and induces apoptosis of colorectal cancer cells Affects gene expression of colon epithelial cells Plays a protective role against colon cancer and colitis Improves the gut barrier function by stimulation of the formation of mucin, antimicrobial peptides, and tight-junction proteins Interacts with the immune system Has anti-inflammatory effects Stimulates the absorption of water and sodium Reduces oxidative stress in the colon **Other effects** Promotes satiety

Changes in the gut microbiota composition have been associated with disturbed gut barrier functions, increased gut permeability, and increased plasma lipopolysaccharide concentrations (i.e., metabolic endotoxemia), which causes low-grade inflammation that triggers the development of obesity and metabolic syndrome (Cani et al., 2008). Also other disorders, such as inflammatory bowel disease (IBD, encompassing Crohn's disease and ulcerative colitis), irritable bowel syndrome (IBS), colorectal cancer, and allergies have been linked to changes in the gut microbiota composition (de Vos and de Vos, 2012; Le Chatelier et al., 2013). During the last years, even associations have been made between the gut microbiota composition and behavioral disorders, such as depression, anxiety disorder, regressive autism, and schizophrenia (Collins et al., 2012; Braniste et al., 2014; Dinan et al., 2015). However, whereas increasing numbers of animal studies provide evidence for cause-and-effect relationships between shifts in gut microbiota composition and certain disorders (as in the case of obesity; Ridaura et al., 2013), it has not been proven yet for humans whether changes in the gut microbiota composition can cause disorders or that these changes are a consequence of the disorders themselves (de Vos and de Vos, 2012).

In recent years, a few distinct members of the human gut microbiota have received particular attention because of their dedicated metabolism and central role in gut homeostasis and because their loss adversely affects the remaining microorganisms and/or host's health. *Bifidobacterium* species are one such bacterial species that fulfill important functions within the human colon (Leahy et al., 2005; Rossi and Amaretti, 2011). Decreased numbers of these species in the colon have been associated with several disorders. Moreover, they have shown to interact with other colon bacteria such as butyrate-producing bacteria by cross-feeding interactions. Furthermore, decreased butyrate concentrations and decreased numbers of butyrate producers in the human colon have been associated with disorders. Therefore, this knowledge has encouraged the development of approaches to stimulate the growth and/or activity of bifidobacteria, i.e., the bifidogenic effect, and butyrate-producing colon bacteria, i.e., the butyrogenic effect, in the human colon. The most prevalent approaches to cause bifidogenic and butyrogenic effects involve the consumption of probiotics and prebiotics.

## Bifidobacteria and butyrate-producing colon bacteria

### *Bifidobacterium* species

#### General aspects

Bifidobacteria are Gram-positive, anaerobic, saccharolytic bacteria that belong to the phylum Actinobacteria; they mainly occur in the gastrointestinal tract of mammals, birds, and insects, but are present in sewage, human breast milk, fermented milk, cheeses, and water kefir too (Bottacini et al., 2014; Khodayar-Pardo et al., 2014; Laureys and De Vuyst, 2014; Laureys et al., 2016). A typical bifidobacterial genome has an average size ranging from 2.0 to 2.8 Mb and is characterized by a high guanine-plus-cytosine content, with numerous genes involved in the uptake and breakdown of carbohydrates from both diet and host origin (Ventura et al., 2014). Bifidobacteria are among the first bacteria to colonize the human gastrointestinal tract and reach their highest proportion in the colon (up to 90% of the total colon microbiota in vaginally delivered breast-fed infants) during the first 12 months of life (Tannock, 2010; Turroni et al., 2012). This abundance significantly decreases over time to <5% in adult subjects and decreases even more in the elderly (Arumugam et al., 2011; Duncan and Flint, 2013). At the time of writing, the *Bifidobacterium* genus comprised 51 species (Euzéby, 1997, 2016; Laureys et al., 2016), among which *Bifidobacterium longum, Bifidobacterium animalis, Bifidobacterium adolescentis, Bifidobacterium bifidum, Bifidobacterium catenulatum, Bifidobacterium pseudocatenulatum, Bifidobacterium breve, Bifidobacterium pseudolongum, Bifidobacterium gallicum, Bifidobacterium angulatum*, and *Bifidobacterium faecale* are encountered in the human colon (Turroni et al., 2009; Ventura et al., 2011; Choi et al., 2014). In general, *B. bifidum* and *B. longum* are the dominant species in infants, whereas *B. adolescentis* and *B. longum* dominate the adult gut microbiota (Turroni et al., 2012). Quantitative PCR analyses of fecal samples of 42 Belgian healthy adults have shown that the fecal microbiota of adults contains between zero and four (with an average of two) different bifidobacterial species, among which *B. longum* (present in 90% of the adults), *B. adolescentis* (present in 79% of the adults), and *B. catenulatum* (present in 38% of the adults) are the most frequently detected species (Ishikawa et al., 2013).

#### Functional role in the colon

From the growing body of scientific evidence associating decreased numbers of bifidobacteria with disorders, it emerges that these species have a disproportionally large impact in the human colon in relation to their relatively low numerical abundance in adults. Hence, a decrease in the relative abundances of *Bifidobacterium* species in the human colon has been associated with antibiotic-associated diarrhea, IBS, IBD, obesity, allergies, and regressive autism (Di Gioia et al., 2014; Grimm et al., 2014). Examples of functions carried out by bifidobacteria include the production and/or liberation of B vitamins, antioxidants, polyphenols, and conjugated linoleic acids; maturation of the immune system during early life and preservation of immune homeostasis during life; preservation of gut barrier functions and protection against pathogens by producing bacteriocins, decreasing luminal pH by the production of acids, and blocking the adhesion of pathogens to the intestinal mucosa (Leahy et al., 2005; Gorissen et al., 2010, 2012; Rossi and Amaretti, 2011; Gagnon et al., 2015). However, these functions are not characteristic for the *Bifidobacterium* genus or certain species, but are rather strain-specific. Another important function of the bifidobacterial genus that contributes to gut homeostasis and host health is the production of acetate and lactate during carbohydrate fermentation, organic acids that in turn can be converted into butyrate by other colon bacteria through cross-feeding interactions (Table 1; De Vuyst and Leroy, 2011; De Vuyst et al., 2014; Rivière et al., 2015).

#### Metabolism

Bifidobacteria display a strictly fermentative metabolism, i.e., they gain energy in the form of ATP by substrate-level phosphorylation during anaerobic carbohydrate breakdown, and play an important role in the human colon with respect to the degradation of carbohydrates that resist digestion and absorption in the upper gastrointestinal tract (Pokusaeva et al., 2011; De Vuyst et al., 2014). Glycoside hydrolases (EC 3.2.1.x) constitute the most important enzyme group that colon bacteria use to degrade poly- and oligosaccharides to fermentable monosaccharides (van den Broek et al., 2008; van den Broek and Voragen, 2008). Compared with the human genome, encoding only 17 glycoside hydrolases for the digestion of food carbohydrates, bifidobacterial genomes possess high numbers of genes encoding these carbohydrases (El Kaoutari et al., 2013). As an example, the genome of *B. longum* NCC2705 contains 56 genes encoding glycoside hydrolases, one gene encoding a carbohydrate esterase (EC 3.1.1.x), but no genes encoding polysaccharide lyases (EC 4.2.2.x; Schell et al., 2002; Lombard et al., 2014). Bifidobacteria are particularly specialized in efficient uptake of short oligosaccharides into the cell, where they are further degraded to monosaccharides, i.e., they display a preferential oligosaccharide metabolism, providing them a competitive advantage toward other colon bacteria that degrade carbohydrates extracellularly (Van der Meulen et al., 2004, 2006b; Falony et al., 2009a; De Vuyst and Leroy, 2011; De Vuyst et al., 2014). About 5% of the total bifidobacterial gene content is dedicated to carbohydrate internalization, through either ATP-binding cassette transporters, permeases, or proton symporters (Ventura et al., 2009). For example, *B. longum* NCC2705 contains 15 genes that encode transport systems that could be involved in the transport of oligosaccharides (Schell et al., 2002; Parche et al., 2007). Several laboratory fermentation studies have shown that bifidobacteria can use various non-digestible carbohydrates as energy sources, encompassing plant-derived carbohydrates [such as resistant starch, pectin, inulin, arabinoxylan (AX), cellulose, and their corresponding oligosaccharides] and host-produced carbohydrates (human milk oligosaccharides and mucin), although this ability is strain-dependent too (Klijn et al., 2005; De Vuyst et al., 2014; Rivière et al., 2014; McLaughlin et al., 2015; Selak et al., 2016).

**Figure 2:**
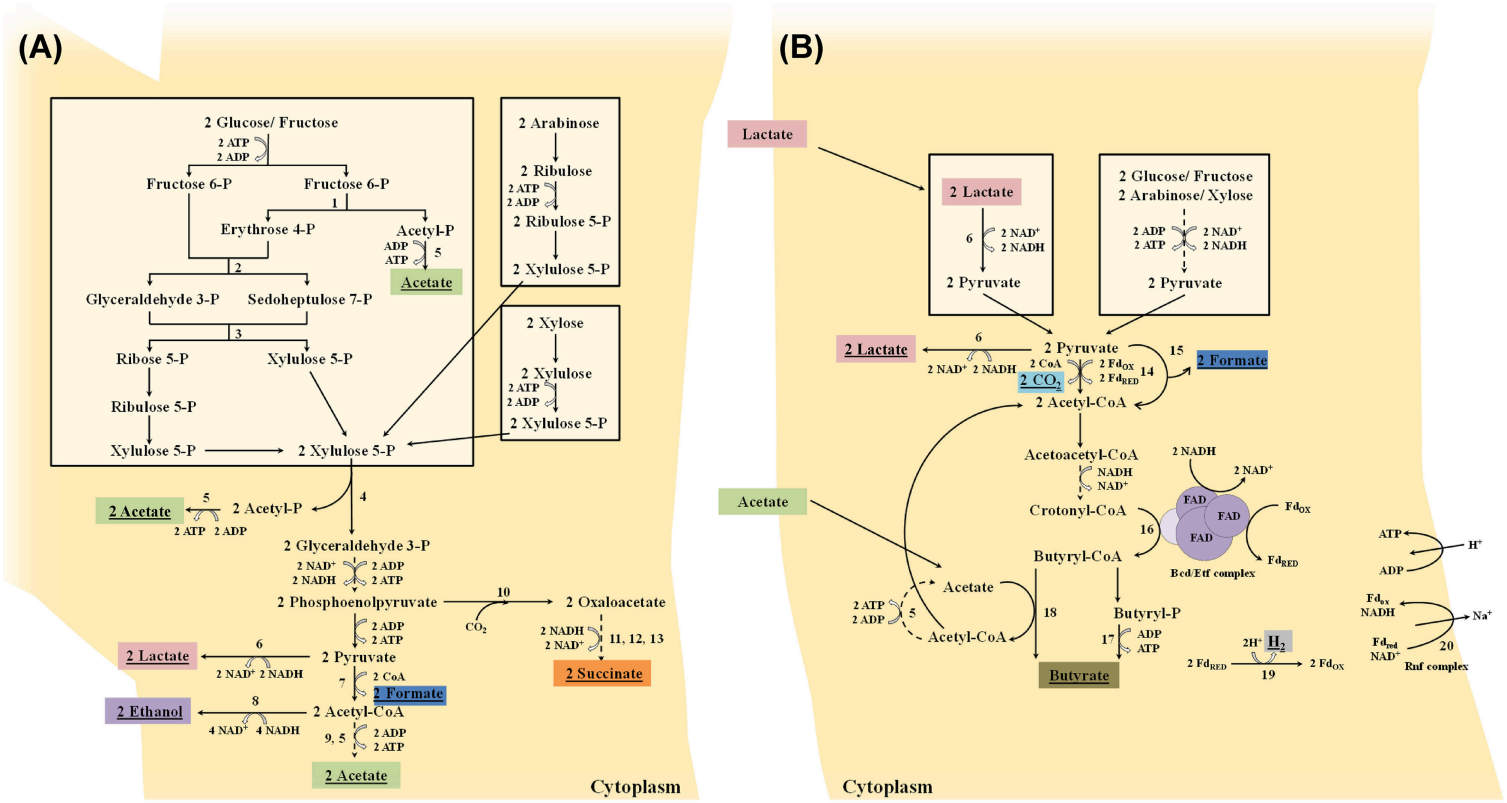
**(A)** Schematic representation of the fermentation of hexoses (glucose and fructose) and pentoses (arabinose and xylose) by bifidobacteria through the fructose 6-phosphate phosphoketolase pathway or bifid shunt. **(B)** Schematic representation of the fermentation of hexoses (glucose and fructose) and pentoses (arabinose and xylose) by butyrate-producing colon bacteria through the Embden-Meyerhof-Parnas pathway and pentose-phosphate pathway, respectively, and of lactate. Dashed lines represent different steps. Underlined metabolites are excreted into the extracellular medium. Fd_ox_, oxidized ferredoxin; Fd_red_, reduced ferredoxin; FAD, flavin adenine dinucleotide; enzymes: 1, fructose 6-phosphate phosphoketolase; 2, transaldolase; 3, transketolase; 4, xylulose 5-phosphate phosphoketolase; 5, acetate kinase; 6, lactate dehydrogenase; 7, formate acetyltransferase; 8, bifunctional aldehyde-alcohol dehydrogenase; 9, phosphotransacetylase; 10, phosphoenolpyruvate carboxylase; 11, malate dehydrogenase; 12, fumarase; 13, succinate dehydrogenase; 14, pyruvate:ferredoxin oxidoreductase; 15, pyruvate-formate lyase; 16, butyryl-CoA dehydrogenase/electron-transferring flavoprotein (Bcd/Etf) complex; 17, butyrate kinase; 18, butyryl-CoA:acetate CoA transferase; 19, ferredoxin hydrogenase; and 20, membrane-bound ferredoxin oxidoreductase (Rnf) complex.

Once internalized into the cytoplasm, hexose monosaccharides (e.g., fructose and glucose) are converted into acetate and lactate by the fructose 6-phosphate phosphoketolase pathway or bifid shunt (De Vuyst et al., 2014). Bifidobacteria will initially cleave, by means of the key enzyme fructose 6-phosphate phosphoketolase, one mole of fructose 6-phosphate into one mole of erythrose 4-phosphate and one mole of acetyl-phosphate (Figure 2A). From erythrose 4-phosphate and an additional mole of fructose 6-phosphate, one mole of ribose 5-phosphate and one mole of xylulose 5-phosphate are formed by the successive action of a transaldolase and a transketolase. Two moles of xylulose 5-phosphate are subsequently converted into two moles of acetyl-phosphate and two moles of glyceraldehyde 3-phosphate by the action of a xylulose 5-phosphate phosphoketolase. These two moles of acetyl-phosphate plus the additional mole of acetyl-phosphate (produced by the fructose 6-phosphate phosphoketolase) are further converted into three moles of acetate by an acetate kinase, which is accompanied by the production of three moles of ATP. The two moles of glyceraldehyde 3-phosphate are oxidized into two moles of pyruvate by enzymes participating in the Embden-Meyerhof-Parnas pathway, which results in an additional production of two moles of ATP. In a last step, pyruvate can be reduced into lactate by means of a lactate dehydrogenase, which is accompanied by NAD^+^ recycling. Thus, when fermenting hexose monosaccharides, acetate and lactate are produced in a theoretical molar ratio of 1.5 and three moles of ATP are produced. Pentose monosaccharides (e.g., arabinose and xylose) can also be shuttled into the bifid shunt by their conversion into xylulose 5-phosphate (Figure 2A). However, this is not accompanied by the production of an additional mole of acetate (and thus no additional mole of ATP) as in the case of hexose fermentation, leading to a final theoretical molar ratio of acetate to lactate of 1.0 and two moles of ATP (Pokusaeva et al., 2011; De Vuyst et al., 2014). However, these theoretical ratios are rarely found during bifidobacterial carbohydrate fermentation, due to the production of formate, acetate, and ethanol from pyruvate instead of lactate (Figure 2A), which depends on the available energy source and its consumption rate (Palframan et al., 2003; Van der Meulen et al., 2004, 2006a,b; Falony et al., 2009b; De Vuyst et al., 2014). The production of formate from pyruvate by a formate acetyltransferase, at the expense of lactate, can be explained by the need for additional ATP production by means of the concomitant production of acetate when bifidobacteria are grown on complex carbohydrates to improve their fitness, despite their lower growth rate compared with simple carbohydrates. Bifidobacteria are also able to produce ethanol from acetyl-CoA with a bifunctional aldehyde-alcohol dehydrogenase, at the expense of acetate, to enable the continuation of pyruvate production by regenerating NAD^+^. This shift in metabolism away from lactate production has been found for the degradation of complex carbohydrates such as inulin-type fructans (ITF; oligofructose and inulin; Van der Meulen et al., 2004; Falony et al., 2009b) and arabinoxylan-oligosaccharides (AXOS; Rivière et al., 2014, 2015). Bifidobacteria can also regenerate NAD^+^ by the production of succinate from oxaloacetate that is in turn formed from phosphoenolpyruvate (Figure 2A; Van der Meulen et al., 2006a).

### Butyrate-producing colon bacterial species

#### General aspects

Gene-targeted approaches to investigate the butyrate-producing bacterial communities of the human gut microbiota have led to the consideration that butyrate-producing colon bacteria form a functional group rather than a monophyletic group. Most butyrate producers in the human colon belong to the Firmicutes phylum and in particular clostridial clusters IV and XIVa (Louis and Flint, 2009; Van den Abbeele et al., 2013a; Vital et al., 2014). Clostridial clusters IV and XIVa butyrate producers are Gram-positive, highly oxygen-sensitive, strictly anaerobic, saccharolytic bacteria. The two most dominant bacterial species in the human colon are *Faecalibacterium prausnitzii* (up to 14% of the total fecal gut microbiota, clostridial cluster IV) and *Eubacterium rectale* (up to 13% of the total fecal gut microbiota, clostridial cluster XIVa), and are expected to have a significant contribution to butyrate production (De Vuyst et al., 2014; Walker et al., 2014). Other important butyrate-producing bacterial species in the human colon are *Roseburia* spp. (clostridial cluster XIVa, such as *Roseburia faecis, Roseburia inulinivorans, Roseburia intestinalis*, and *Roseburia hominis*), *Eubacterium* spp. (clostridial cluster XIVa, such as *Eubacterium hallii*), *Anaerostipes* spp. (clostridial cluster XIVa, such as *Anaerostipes butyraticus, Anaerostipes caccae*, and *Anaerostipes hadrus*), and *Butyricicoccus pullicaecorum* (clostridial cluster IV). Some of these species (such as *E. rectale, F. prausnitzii*, and *R. intestinalis*) preferentially colonize the mucus layer, and consequently increase the butyrate bioavailability for colon epithelial cells, whereas other species such as *A. caccae* mainly occur in the lumen of the colon (El Aidy et al., 2013; Van den Abbeele et al., 2013a). In contrast to bifidobacteria, clostridial clusters IV and XIVa do not directly colonize the colon in high quantities after birth. In the case of *F. prausnitzii*, it has been shown that fecal numbers in infants younger than 6 months are undetectable, slightly increase between the age of 6 and 24 months, then suddenly increase to reach a peak during late childhood and adolescence, and finally decrease again during adulthood and especially in the elderly (Miquel et al., 2014).

#### Functional role in the colon

Clostridial clusters IV and XIVa have gained a lot of attention during the last years because of their contribution to gut homeostasis, by preserving gut barrier functions and exerting immunomodulatory and anti-inflammatory properties (Velasquez-Manoff, 2015). In addition to the beneficial properties of the butyrate produced (Table 1), *F. prausnitzii* produces anti-inflammatory peptides blocking nuclear factor NF-κB activation and cytokine IL-8 production in mice, which provide protection against chemically induced colitis (Qiu et al., 2013; Quévrain et al., 2016). Several studies have shown that the abundance of *B. pullicaecorum, E. rectale, F. prausnitzii*, and/or *R. intestinalis* is markedly decreased in IBD patients (Morgan et al., 2012; Eeckhaut et al., 2013; Gevers et al., 2014) and that such patients have lower concentrations of butyrate in their feces than healthy individuals (Marchesi et al., 2007; Nemoto et al., 2012). Less butyrate producers were also found in patients with colorectal cancer (Wu et al., 2013). Therefore, methods are being searched to stimulate butyrate-producing human colon bacterial species by diet (prebiotic approach) or by administering these bacteria orally (probiotic approach). In medical applications, pure butyrate by means of tablets or rectal enemas is used as a therapeutic agent for IBD treatment (Geirnaert et al., 2014).

#### Metabolism

Like bifidobacteria, members of clostridial clusters IV and XIVa carry out a fermentative metabolism and are often able to degrade a wide range of non-digestible carbohydrates in the human colon anaerobically, encompassing resistant starch, ITF, xylo-oligosaccharides (XOS), and AXOS (Falony et al., 2009c; Louis and Flint, 2009; Scott et al., 2014; Rivière et al., 2015; Moens et al., 2016). As an example, the genome of *E. rectale* ATCC 33656 encodes 52 glycoside hydrolases, encompassing β-fructofuranosidases, α-arabinofuranosidases, β-xylosidases, exo-oligoxylanases, α-amylases, α- and β-glucosidases, α- and β-galactosidases, and cellulases (Lombard et al., 2014). However, inter-genus variations have been found within clostridial clusters IV and XIVa, as not all species and even strains within one species can consume complex carbohydrates to the same extent (Falony et al., 2009c; Scott et al., 2014; Moens et al., 2016). Most of the butyrate-producing colon bacteria use a non-preferential extracellular degradation mechanism for the breakdown of oligo- and polysaccharides, with the release of monosaccharides into the extracellular medium. As illustrated during laboratory batch fermentation experiments, co-cultivation of such butyrate-producing bacteria with bifidobacteria that have a preferential carbohydrate degradation mechanism, can comprise the competitiveness of the butyrate-producing colon bacteria (Falony et al., 2006, 2009c; De Vuyst et al., 2014). For instance, the percentage of oligofructose that was consumed by *F. prausnitzii* DSM 17677^T^ when co-cultivated with different bifidobacterial strains decreased with an increasing ITF degradation capacity of the latter (Moens et al., 2016).

Once internalized into the cytoplasm, hexoses and pentoses are degraded to pyruvate by the Embden-Meyerhof-Parnas pathway or pentose phosphate pathway, respectively. Like other fermentative bacteria, clostridial clusters IV and XIVa butyrate producers possess several alternative pathways to form different end-metabolites from pyruvate, depending on the bacterial species, carbohydrate source, hydrogen gas pressure, and necessity of redox balancing. Besides butyrate, they can form lactate, formate, hydrogen gas, and carbon dioxide (Figure 2B). Pyruvate can get reduced into lactate by means of a lactate dehydrogenase, which is accompanied by NAD^+^ recycling (for instance *R. inulinivorans* and *E. rectale*; Falony et al., 2009c; Rivière et al., 2015; Moens et al., 2016). The production of butyrate from pyruvate involves the conversion of pyruvate into acetyl-CoA by a pyruvate:ferredoxin oxidoreductase, with the reduction of ferredoxin and production of carbon dioxide (for instance most clostridial clusters IV and XIVa butyrate-producing colon bacteria; Falony et al., 2009c; Moens et al., 2016) and/or by a pyruvate-formate lyase with the formation of formate (for instance *F. prausnitzii* and *E. rectale*; Rivière et al., 2015; Moens et al., 2016). Two moles of acetyl-CoA are then converted via a four-step pathway into butyryl-CoA, in which the last step is carried out by a butyryl-CoA dehydrogenase/electron-transferring flavoprotein complex that catalyzes the NADH + H^+^-dependent reduction of crotonyl-CoA coupled to the reduction of ferredoxin. The final step from butyryl-CoA to butyrate is either catalyzed by a butyrate kinase (after phosphorylation of butyryl-CoA) or a butyryl-CoA:acetate CoA transferase (Falony et al., 2009c; Louis and Flint, 2009; Mahowald et al., 2009; De Vuyst and Leroy, 2011; Moens et al., 2016). Only a few butyrate-producing colon bacteria, encompassing *Clostridium butyricum, Coprococcus eutactus*, and *Coprococcus comes*, are known to use a butyrate kinase to produce butyrate (Louis and Flint, 2009; Vital et al., 2014). The butyryl-CoA:acetate CoA transferase step involves the consumption of external acetate (coming from for instance bifidobacterial carbohydrate breakdown through cross-feeding) as a co-substrate, thereby producing acetyl-CoA and butyrate. The acetyl-CoA produced can be converted via acetyl-phosphate into acetate, with the production of ATP, by acetate kinase, or recycled into the four-step pathway mentioned above (Falony et al., 2009c). The reduced ferredoxin can be reoxidized via a ferredoxin hydrogenase, with the concomitant production of H_2_, and/or via a membrane-bound ferredoxin oxidoreductase (Rnf) complex, without production of H_2_, but with the generation of a proton-motive force that allows additional ATP production (for instance *R. inulinivorans* and *F. prausnitzii*; Falony et al., 2009c; Moens et al., 2016). The production of butyrate thus not only leads to regeneration of NAD^+^ from NADH + H^+^ produced in the upper parts of the carbohydrate degradation pathways for ATP production, but also leads to additional ATP production (Falony et al., 2009c; Louis and Flint, 2009; Mahowald et al., 2009; De Vuyst and Leroy, 2011). Some butyrate producers, encompassing *A. caccae, A. butyraticus, A. hadrus*, and *E. hallii*, can produce butyrate from lactate instead of carbohydrates (Figure 2B; Duncan et al., 2004; Falony et al., 2006, 2009c; Belenguer et al., 2011; De Vuyst and Leroy, 2011; De Vuyst et al., 2014).

## Stimulation of bifidobacteria and butyrate-producing colon bacteria

Since decreased numbers of *Bifidobacterium* species and butyrate-producing bacterial species in the human colon have been reported in patients with diverse disorders and because the SCFAs produced by these species have beneficial effects (Table 1), these bacteria are potential candidates to be stimulated in the colon to prevent and restore a disturbed gut homeostasis. The most prevalent strategies to stimulate bifidobacteria and butyrate-producing colon bacteria in the human colon involve the consumption of probiotics and prebiotics (Scott et al., 2015).

### Probiotics

According to the international scientific association for probiotics and prebiotics (ISAPP), probiotics are defined as “live microorganisms that, when administered in adequate amounts, confer a health benefit on the host” (Hill et al., 2014). Selected strains of *Bifidobacterium* species are commonly used probiotics and are added to food supplements and foods (especially dairy products). The oral consumption of bifidobacteria has been associated with beneficial effects for different digestive problems and disorders, encompassing acceleration of the gut transit time; improvement of lactose intolerance; prevention of antibiotic-associated diarrhea and necrotizing enterocolitis (in pre-term infants that usually harbor reduced numbers of bifidobacteria); and alleviation of IBS and IBD symptoms (Leahy et al., 2005; Di Gioia et al., 2014; Tojo et al., 2014; Saez-Lara et al., 2015). Also, evidence continues to emerge that bifidobacteria influence immune responses and hence may enhance resistance to infections and allergies (Di Gioia et al., 2014; Frei et al., 2015). Further, bifidobacteria display anti-inflammatory effects and negatively correlate with metabolic endotoxemia (Everard and Cani, 2013). Moreover, interest is growing to use bifidobacterial strains (such as *Bifidobacterium infantis* 35624) as psychobiotics, which are “live organisms that, when ingested in adequate amounts, produce a health benefit in patients suffering from psychiatric illness” (Dinan et al., 2013, 2015). The health effects that bifidobacteria exert are of course strain-related; some bifidobacterial strains are effective, whereas others are not. Moreover, the probiotic health benefits are probably not caused by the bifidobacterial strains consumed solely, but are rather the result of interactions with the resident gut microbiota (Cani and Van Hul, 2015; Scott et al., 2015). Indeed, a recent metagenomic and metatranscriptomic study of feces of 12 healthy individuals has shown that the oral administration of the probiotic strain *Lactobacillus rhamnosus* GG significantly changes the activity of the resident gut microbiota, without influencing the gut microbiota composition itself (Eloe-Fadrosh et al., 2015). Especially genes involved in adhesion, chemotaxis, and/or motility of *Bifidobacterium* spp., *Eubacterium* spp., and *Roseburia* spp. are overexpressed during probiotic consumption, suggesting that the consumption of the probiotic strain promotes interactions between the resident gut microbiota and the host. Nowadays, there is also a growing interest toward the use of other bacterial strains as probiotics, such as *Akkermansia muciniphila* and butyrate-producing colon bacteria, encompassing *B. pullicaecorum, E. rectale, F. prausnitzii*, and *Roseburia* spp. (Marteau, 2013; Geirnaert et al., 2014; Cani and Van Hul, 2015; Scott et al., 2015). For example, the oral administration of *B. pullicaecorum* 25-3^T^ and *F. prausnitzii* A2-165 in rodents has shown attenuation of chemically induced colitis (Eeckhaut et al., 2013, 2014; Martín et al., 2015). However, whether these strict anaerobic colon bacteria can survive the harsh industrial production steps and deal with the regulatory hurdles (as these bacteria have no history of safe use) will partly determine their application as probiotics in the future human diet (Figueroa-González et al., 2011; Gosálbez and Ramón, 2015; Kumar et al., 2015; Scott et al., 2015).

Since the implementation of EU legislation on health claims in 2009, no health claims for probiotics in foods have been approved by the European Food Safety Authority (EFSA) neither can the term probiotic further be used as a food label in Europe (Glanville et al., 2015). The only approved health claim is the benefit on lactose digestion when consuming live *Lactobacillus delbrueckii* subsp. *bulgaricus* and *Streptococcus thermophilus* strains present in yogurt or fermented milk (EFSA, 2010).

In severe cases of a disturbed gut homeostasis, whereby probiotic treatments do not suffice, the gut microbiota can be restored by transplanting the complete fecal microbiota from a healthy donor into a diseased person. However, the ISAPP recommends that fecal microbiota transplantations (FMTs) should not be considered as probiotics, as they are uncharacterized mixtures of strains (Hill et al., 2014). FMTs have shown to be very effective for curing *Clostridium difficile* infections, although they have ambiguous outcomes for IBD and IBS (Aroniadis and Brandt, 2014; Pamer, 2014). Furthermore, a step-up FMT strategy has been proposed to treat Crohn's disease and ulcerative colitis, which consists of a FMT, followed by additional FMT steps or standard IBD medications depending on the patient's clinical response to the treatment (Cui et al., 2016). Also, it has been shown that patients with metabolic syndrome display improved insulin sensitivity after being treated with fecal microbiota of healthy individuals (Vrieze et al., 2012). These patients possess increased numbers of butyrate-producing colon bacteria and decreased numbers of Gram-negative bacteria after a FMT. Studies are being performed to see whether FMTs can also cure non-gastrointestinal disorders, such as allergies and behavioral disorders (Xu et al., 2015). However, up to now, few fecal transplants have been performed, as the selection of healthy fecal donors requires a thorough examination to avoid the transfer of pathogens and gut microbiota-associated disorders (Kapel et al., 2014). Therefore, new approaches are being searched to transplant well-defined mixtures of bacteria (de Vos, 2013; Van den Abbeele et al., 2013b). However, an additional challenge in selecting an appropriate healthy donor or bacterial synthetic community is that, despite the large amount of information about the composition and diversity of the human gut microbiota, it is difficult (if not impossible) to define a healthy gut microbiota composition, as each healthy individual harbors a unique gut microbiota (de Vos and de Vos, 2012; Faith et al., 2013; Li et al., 2016).

### Prebiotics

#### General

Another strategy to increase bifidobacteria and butyrate-producing bacteria in the human colon is through the consumption of prebiotics, which are defined according to the ISAPP as “a selectively fermented ingredient that results in specific changes in the composition and/or activity of the gastrointestinal microbiota, thus conferring benefit(s) upon host health” (Gibson et al., 2010). To date, all well-known prebiotics are carbohydrates, although other compounds such as, for instance, polyphenols may display prebiotic properties as well (Bindels et al., 2015). Compared with probiotics, prebiotics are more stable and thus can easily be added to foods, such as yogurts, biscuits, breads, cereals, spreads, ice creams, and drinks (Gibson et al., 2010). The criteria for classifying a compound as a prebiotic have been listed as (i) resistance to gastric acidity, hydrolysis by mammalian digestive enzymes, and gastrointestinal absorption; (ii) fermentation by intestinal microbiota; and (iii) selective stimulation of the growth and/or activity of intestinal bacteria associated with health and well-being (Gibson et al., 2004). In the past, the impact of the consumption of prebiotics on the gut microbiota composition was mainly studied regarding species of *Bifidobacterium* and *Lactobacillus* (Verbeke, 2014). However, recent community-wide analyses of the gut microbiota show that prebiotics are not that selective as previously assumed, and that they stimulate other bacteria too (Bindels et al., 2015). It has indeed been shown that butyrate-producing colon bacteria, such as *E. rectale, F. prausnitzii*, and *Roseburia* spp., can consume prebiotics such as ITF (Falony et al., 2006, 2009c; Rivière et al., 2015; Moens et al., 2016). Also, the consumption of oligofructose changes the relative abundance of 102 bacterial taxa in mice, of which 16 display a more than 10-fold decrease or increase in abundance (Everard et al., 2011). Therefore, Bindels et al. (2015) proposed to define a prebiotic as “a non-digestible compound that, through its metabolization by microorganisms in the gut, modulates composition and/or activity of the gut microbiota, thus conferring a beneficial physiological effect on the host.” Alternatively, the definition of prebiotics has been challenged over time not only according to scientific considerations but also due to its importance for regulators, industry, and consumers (Hutkins et al., 2016). As for probiotics, the term prebiotic cannot be used as a health claim on food products in Europe (Salminen and van Loveren, 2012). Some claims exist for the term fiber (EFSA, 2011a,b, 2015), but not all fibers are prebiotics, whereby the latter are distinguished from the former by the selectivity of their fermentation (Slavin, 2013; Hutkins et al., 2016; Verspreet et al., 2016).

Examples of prebiotic non-digestible carbohydrates that are bifidogenic include poly- and oligosaccharides containing fructose (and a terminal glucose) as in ITF, galactose and glucose (as in galacto-oligosaccharides), glucose (as in isomalto-oligosaccharides), galactose and fructose (as in lactulose), xylose (as in XOS), and arabinose and xylose (as in AX and AXOS) (Roberfroid, 2005; Macfarlane et al., 2008; Broekaert et al., 2011; De Vuyst and Leroy, 2011; De Vuyst et al., 2014). Whereas, in the past the target genus for prebiotic stimulation was *Bifidobacterium* (Gibson et al., 2010), today new prebiotics are searched to stimulate other beneficial bacterial species in the human colon such as butyrate producers. Of special interest are prebiotics that cause both a bifidogenic effect and a butyrogenic effect. ITF, AX, and AXOS are such prebiotics that stimulate both bifidobacteria and the production of butyrate (Falony et al., 2006, 2009b,c; De Vuyst and Leroy, 2011; De Vuyst et al., 2014; Rivière et al., 2015).

#### ITF as an example of well-known prebiotics

**Figure 3:**
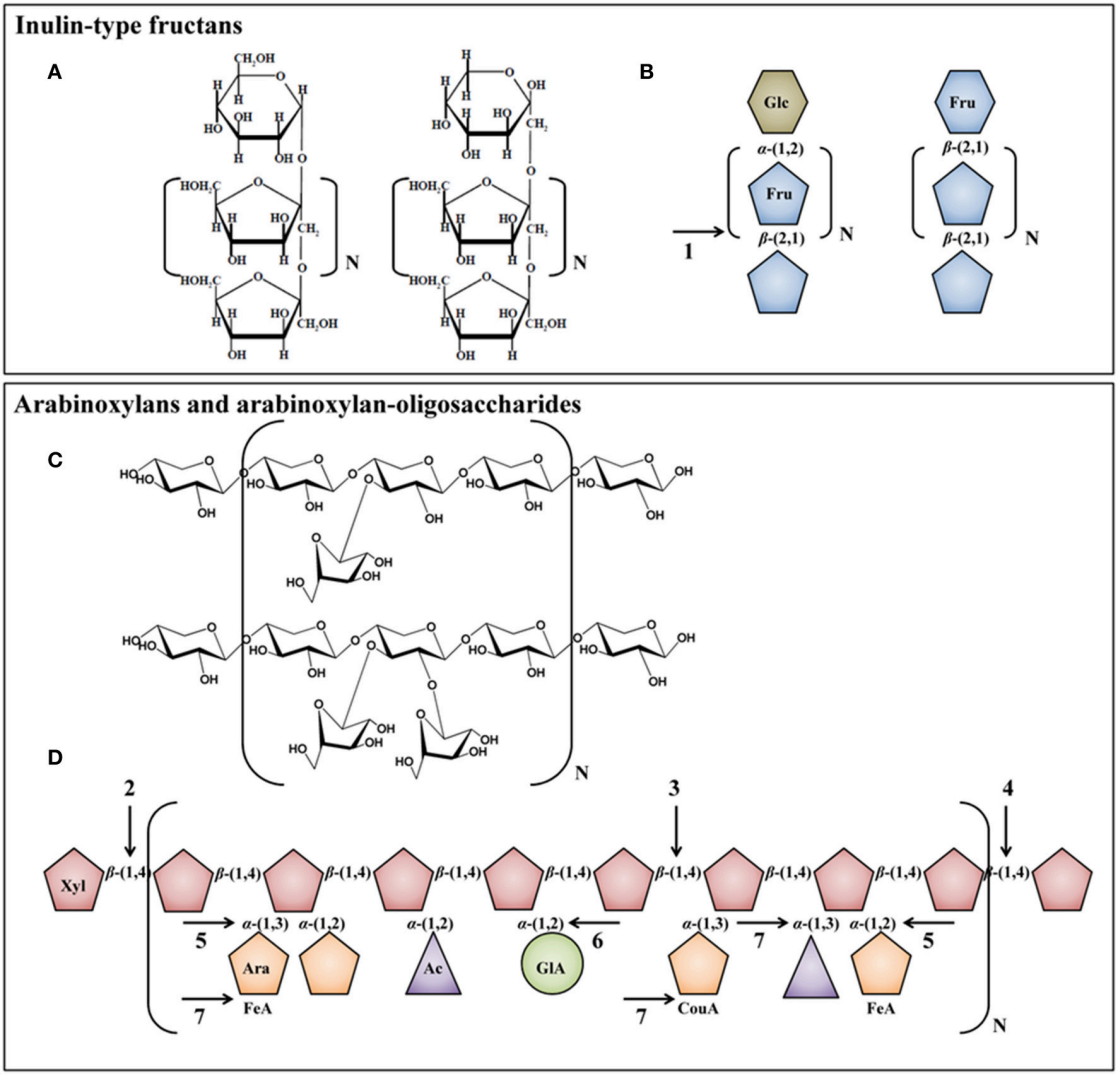
**Chemical structures [(A) and (C)] and schematic representations [(B) and (D)] of ITF, AX, and AXOS molecules**. Glc, glucose; Fru, fructose; Xyl, xylose; Ara, arabinose; FeA, ferulic acid; Ac, acetyl group; GlA, glucuronic acid; CouA, *p*-coumaric acid. Arrows indicate possible hydrolysis of the carbohydrates by bacterial enzymes present in the human colon: 1, β-fructofuranosidase; 2, β-xylosidase; 3, β-endoxylanase; 4, exo-oligoxylanase; 5, α-arabinofuranosidase; 6, α-glucuronidase; and 7, esterase.

Inulin naturally occurs in fruits and plants such as chicory roots, wheat, onion, banana, garlic, and leek, but is generally extracted from chicory roots on an industrial scale (Roberfroid, 2007). Inulin consists of a linear backbone of β-(2 → 1)-linked fructose monomers with a degree of polymerization (DP) between 2 and 65 (average DP of 10), which is often linked to a terminal glucose monomer by an α-(1 → 2)-glycosidic bond (Figures 3A,B). Oligofructose is derived from native inulin by partial enzymatic hydrolysis with an inulinase and has a DP that varies between 2 and 8 (average DP of 4). Given the relative simple structures of ITF, only few bacterial enzymes are required for their degradation in the human colon, encompassing enzymes belonging to the β-fructofuranosidase (EC 3.2.1.26) superfamily that cleave terminal fructose residues from the non-reducing ends of the fructose polymers (Figure 3B; Scott et al., 2011). Several β-fructofuranosidases have been isolated and characterized in colon bacteria, for instance in *Bifidobacterium* species (Warchol et al., 2002; Ehrmann et al., 2003; Omori et al., 2010; Jedrzejczak-Krzepkowska et al., 2011) and *R. inulinivorans* (Scott et al., 2011). Examples of beneficial effects of the consumption of ITF include increased stool frequency, increased colonic absorption of dietary minerals (calcium and magnesium), decreased proteolytic activity, and increased secretion of satiety hormones (Schaafsma and Slavin, 2015).

**Figure 4:**
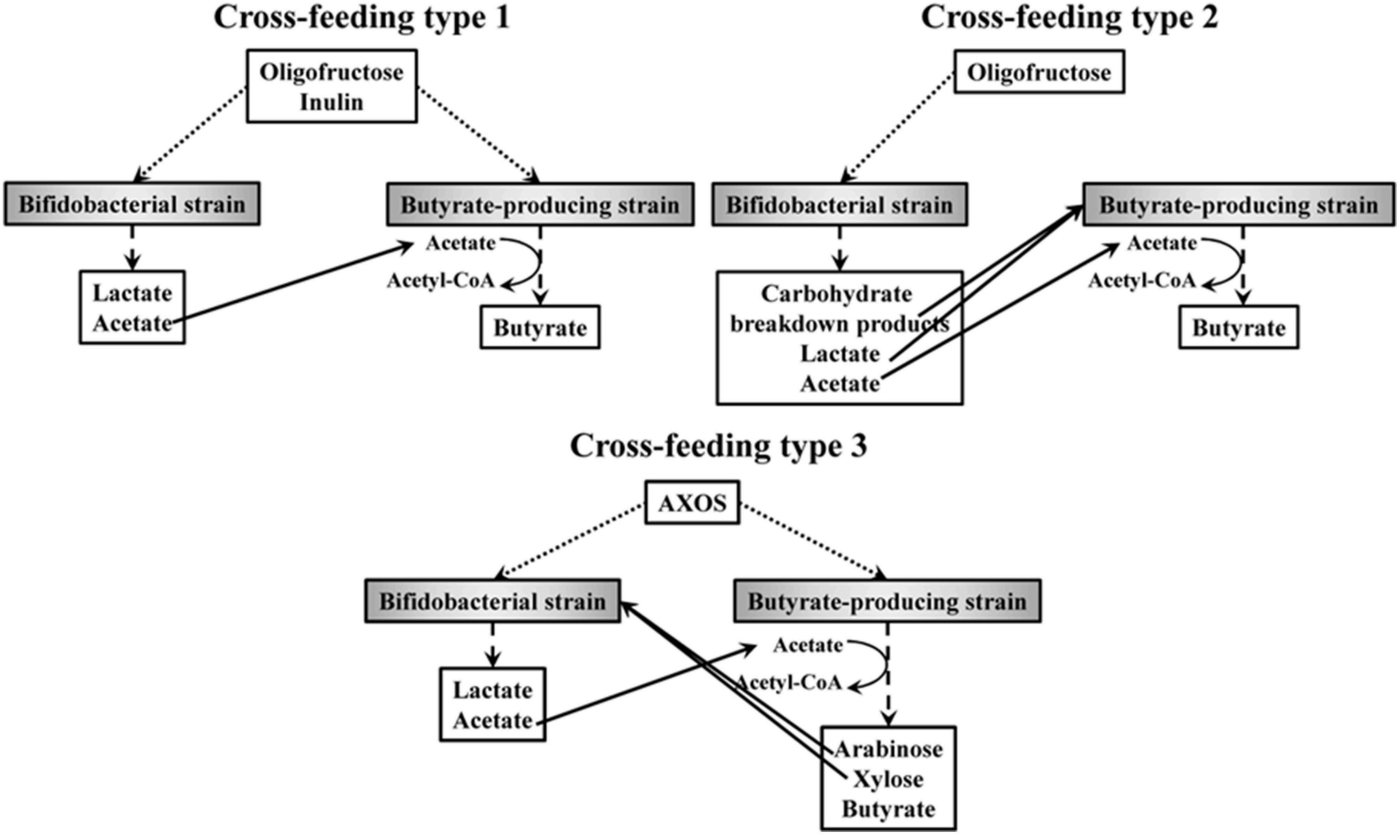
**Different types of cross-feeding that can take place between *Bifidobacterium* spp. and species of butyrate-producing colon bacteria in the human colon**. Arrows indicate consumption of oligofructose, inulin, and AXOS (^…..^), production of carbohydrate breakdown products and/or metabolic end-products (- - -), and cross-feeding interactions between the bifidobacterial and butyrate-producing strains (—).

ITF belong to the most studied prebiotics and their bifidogenic and butyrogenic effects have been well established in various studies (De Vuyst and Leroy, 2011; De Vuyst et al., 2014). For instance, it has been shown that not all bifidobacterial strains benefit in the same way from the presence of ITF in the human colon. A comparative statistical study of 18 bifidobacterial strains, belonging to 10 different species and coming from different donors and origins, has shown the existence of four different clusters of strains differing in their mechanisms and capabilities to degrade ITF (Falony et al., 2009b). Some strains only consume fructose (cluster A), whereas others consume both fructose and oligofructose, mainly short oligosaccharides (DP up to seven) after import into the cell, i.e., they display a preferential metabolism (cluster B). Certain strains degrade both oligofructose and inulin (short chain length fractions only) extracellularly, accompanied with the release of fructose into the extracellular medium, i.e., they display a non-preferential metabolism (clusters C and D). A recent study of 190 bifidobacterial strains isolated from different donors and colon regions has shown that these ITF degradation fingerprints are not correlated with the region in the intestine, suggesting that the degradation of ITF is uniform along the human intestine (Selak et al., 2016). Yet, intra-species variability in ITF degradation capacity indicates strain-specific variations. Moreover, within one colon region bifidobacterial strains with different ITF degradation mechanisms occur, which suggests cooperation for the degradation of ITF in the colon, with opportunities for cross-feeding on strain and/or species level. Similar cross-feeding between bifidobacterial strains with complementary degradation mechanisms has also been shown for starch, xylan, and mucin glycoproteins (Egan et al., 2014; Turroni et al., 2015). Also, it has been shown that the consumption of ITF, the bifidogenic effect, and the butyrogenic effect are linked to each other, because of cross-feeding interactions between bifidobacteria and butyrate-producing colon bacteria (Figure 4; Belenguer et al., 2006; Falony et al., 2006, 2009c; Moens et al., 2016). As an end-metabolite of the bifid shunt and a co-substrate for the production of butyrate (Section AX and AXOS as an Example of Interesting Prebiotics), acetate plays a key role in cross-feeding interactions between bifidobacteria and butyrate-producing colon bacteria in the human colon. In a first type of cross-feeding, both the bifidobacterial and butyrate-producing strains consume ITF (Figure 4). The consumption of ITF by bifidobacteria provides butyrate-producing colon bacteria with exogenous acetate that is used as a co-substrate to produce butyrate by growing on ITF simultaneously [which is, for instance, the case for *R. intestinalis* DSM 14610 (Falony et al., 2006), *R. inulinivorans* DSM 16841 (Falony et al., 2009c), and *F. prausnitzii* DSM 17677^T^ (Moens et al., 2016)]. However, such cross-feeding interactions can be either a pure commensal or beneficial relationship between these bacteria or can be dominated by competition, depending on the ITF degradation capacities of the bifidobacterial strains involved (Moens et al., 2016). A second type of cross-feeding takes place between bifidobacteria that consume ITF, and concomitantly produce acetate, and acetate-consuming butyrate-producing colon bacteria that are not able to degrade ITF (Figure 4). Instead of ITF, the latter bacteria consume carbohydrate breakdown products (short-chain oligosaccharides) liberated by the bifidobacterial strain (which is, for instance, the case for *R. hominis* DSM 16839; Belenguer et al., 2006) or lactate (for instance *E. hallii* DSM 17630; Belenguer et al., 2006, and *A. caccae* DSM 14662; Falony et al., 2006).

#### AX and AXOS as an example of interesting prebiotics

##### Physiological effects

Growing interest is devoted to complex non-digestible carbohydrates that ferment slowly and thereby cause bifidogenic and butyrogenic effects along the entire length of the human colon. AX and AXOS, as a broad class of heteropolysaccharides and -oligosaccharides with complex varying structures (Figures 3C,D), belong to these slow-fermenting carbohydrates and hence are able to decrease the production of bacterial toxic metabolites originating from protein and lipid metabolism in the distal colon (Section Bifidobacterium Species; Van Craeyveld et al., 2008; Grootaert et al., 2009; Sanchez et al., 2009; Neyrinck et al., 2011). This is to be explained by a stimulation of saccharolytic activities, an increase in SCFA production, and a lowering of the luminal pH in the distal part of the colon, where carbohydrates are rare and proteolytic bacteria, such as *Bacteroides* spp., are otherwise favored (Duncan et al., 2009). Examples of additional potential benefits of the consumption of AX and AXOS for human health include improved mineral (calcium and magnesium) absorption; increased stool frequency and improved stool consistency; reduced post-prandial glycemic response; reduced blood cholesterol levels; and increased antioxidant capacity (Grootaert et al., 2007; Broekaert et al., 2011; Damen et al., 2011; Mendis and Simsek, 2013). Moreover, the consumption of AXOS, with the increase of bifidobacterial numbers as a result, may help to restore gut barrier functions and cure metabolic endotoxemia in mice (Neyrinck et al., 2012).

##### Occurrence, structural properties, and degradation

AX naturally occur in the endosperm and bran (pericarp, testa, and aleuron layer) of cereal grains such as wheat, rye, rice, barley, oat, and sorghum, but in varying quantities, depending on the cereal species and the location within the cereal kernel (Izydorczyk and Biliaderis, 2006). For instance, the endosperm of wheat kernels contains *ca*. 2% of AX, whereas the pericarp contains *ca*. 38% of AX (Benamrouche et al., 2002; Maes and Delcour, 2002). AX consist of a linear backbone of 1500 to 15,000 β-(1 → 4)-linked xylose monomers, which can randomly be substituted with arabinose monomers on the C-(O)-2 or C-(O)-3 positions (monosubstituted) or on both positions (disubstituted; Figures 3C,D; Izydorczyk and Biliaderis, 1995). Distribution patterns of arabinose substituents on the xylose backbone are not regular for wheat AX; highly branched regions are interlinked by sequences of contiguous non-substituted xylose residues (Gruppen et al., 1993). The number of arabinose substituents bound to the xylose backbone is expressed as the arabinose/xylose ratio (A/X) and depends on the cereal species and the location within the kernel. For instance, in the pericarp, testa, aleuron layer, and endosperm of wheat kernels, different A/Xs are found, namely *ca*. 1.0, 0.1, 0.4, and 0.5, respectively (Izydorczyk and Biliaderis, 1995; Antoine et al., 2003; Barron et al., 2007). The fermentability of AX and AXOS in the human colon is strongly influenced by the complexity of the AXOS molecules and decreases with increasing DP and increasing A/X (Van Craeyveld et al., 2008; Pollet et al., 2012). Additionally, xylose residues can be esterified with glucuronic acid and acetyl groups, whereas arabinose residues can be esterified with ferulic acid and *p*-coumaric acid, although in low numbers (Figure 3D; Izydorczyk and Biliaderis, 1995). These esterifications are of health and physicochemical importance, since ferulic acid and *p*-coumaric acid are antioxidants and potent cross-linking sites for attachment to other AX chains (Bunzel et al., 2001; Ou and Sun, 2014). The presence of feruloylated and diferuloylated arabinose substituents reduces the fermentability of AX and AXOS (Hopkins et al., 2003; Snelders et al., 2014). As cereal whole grains only contain low concentrations of AX (varying between 1.8% of AX in sorghum and 12.1% of AX in rye; Izydorczyk and Biliaderis, 2006), and thus the overall intake of AX is low (especially in modern Western-type diets with high intakes of refined cereal products), AX can be extracted from cereal grains and added to food products in higher concentrations (Broekaert et al., 2009). On an industrial scale, AX are usually extracted from wheat bran that is available in large quantities in Europe (Swennen et al., 2006). AXOS, the hydrolysis products of AX, are formed not only in processed cereal-based food products such as bread and beer (Courtin et al., 2009; Broekaert et al., 2011), but can also be produced on an industrial scale by the enzymatic cleavage of AX with β-endoxylanases (Broekaert et al., 2009, 2011). This results in various substituted molecules (i.e., AXOS) and non-substituted molecules (i.e., XOS), differing in DP and A/X.

Given their complex structures, the degradation of AX and AXOS in the human colon requires the cooperative action of debranching and depolymerizing bacterial carbohydrate-active enzymes, encompassing β-endoxylanases (EC 3.2.1.8) that cleave AX into AXOS and XOS; β-xylosidases (EC 3.2.1.37) that cleave terminal xylose residues from the non-reducing ends of the xylose backbones; exo-oligoxylanases (EC 3.2.1.156) that release terminal xylose residues from the reducing ends of the xylose backbones; and α-arabinofuranosidases (EC 3.2.1.55) that remove arabinose substituents from the xylose backbones (Figure 3D). Additional enzymes are needed to cleave glucuronic acid [i.e., α-glucuronidase (EC 3.2.1.139)], ferulic acid [i.e., ferulic acid esterase (EC 3.1.1.73)], acetyl groups [i.e., acetyl xylan esterase (EC 3.1.1.72)], and *p*-coumaric acid [i.e., *p*-coumaric acid esterase (EC 3.1.1.-)] from AXOS (Figure 3D; Dodd and Cann, 2009; Lagaert et al., 2014).

To date, AX and AXOS fall under the definition of dietary fiber (European Commission, 2008; Snelders et al., 2014) but are not considered as prebiotics by the EFSA, although they meet the three criteria of prebiotics (see above; Broekaert et al., 2011). AX and AXOS are neither digested nor absorbed in the upper gastrointestinal tract and reach the human colon intact, where they are fermented by the resident colon bacteria and cause bifidogenic and butyrogenic effects (Table 2). However, as is also the case for other prebiotics, the selective stimulation criterion can be questioned. Several *in vivo* and *in vitro* studies have shown that AX and AXOS stimulate, besides bifidobacteria and butyrate-producing colon bacteria, other saccharolytic colon bacteria too, such as *Bacteroides* spp. and *Lactobacillus* spp. (Table 2). Moreover, a propionogenic effect is supposed to occur. A few studies have shown that AX and AXOS especially stimulate the production of propionate (Table 2; Hopkins et al., 2003; Van den Abbeele et al., 2011; Pollet et al., 2012). For instance, the mucin-consuming propionate-producing *A. muciniphila* is stimulated in the colon of humanized rats fed with long-chain AX (Table 2; Van den Abbeele et al., 2011). Whether this is a direct or indirect effect is not known yet.

**Table 2:** **Overview of *in vitro* and *in vivo* studies of AX and AXOS**.

**Substrate (avDP-A/X) supplementation^+^**	**Time**	***In vitro/In vivo***	**Significant concentration shift**^+^		**Method microbial characterization^+^**	**Significant bacterial shift**^+^		**References**
			**Butyrate**	**Propionate**		**Increase of**	**Decrease of**	
AXOS (Nd-0.87) 13 g day^−1^	3 w	*In vivo* Humans	↑ Most	↑	Nd	Nd	Nd	Gråsten et al., 2003
AX (Nd-0.51) 10 g L^−1^	48 h	*In vitro* batch fermentation (human fecal inoculum)	↑	↑ Most	16S rRNA probe hybridization	*Bacteroides-Prevotella-Porphyromonas* spp.	~	Hopkins et al., 2003
AX-66 kDa (Nd-0.40) AX-278 kDa (Nd-0.61) AX-354 kDa (Nd-0.61) 1% (m v^−1^)	12 h	*In vitro* batch fermentation (human fecal inoculum)	↑ Most Especially AX-66 kDa	↑	Fluorescent *in situ* hybridization (FISH)	*Bifidobacterium* spp., *Lactobacillus* spp., and *Bacteroides* spp. *Clostridium coccoides-Eubacterium rectale* spp. (especially AX-66 kDa)	~	Hughes et al., 2007
AXOS (61-0.58) (12-0.69) (15-0.27) (5-0.27) (3-0.26) 4% (m m^−1^)	2 w	*In vivo* Rats	↑ Only for AXOS (5-0.27) and (3-0.26) in colon	~	qPCR	*Bifidobacterium* spp. [only for AXOS (5-0.27, 3-0.26) in cecum]	~	Van Craeyveld et al., 2008
AXOS (15-0.27) 3 g L^−1^	3 w	*In vitro* SHIME® (human fecal inoculum)	↓ In proximal colon vessel ↑ In transverse colon vessel	↓ In proximal colon vessel ↑ Most in transverse colon vessel	qPCR	~	*Roseburia* spp. (in proximal colon vessel)	Grootaert et al., 2009
AXOS (29-0.30) 3 g L^−1^	3 w	*In vitro* SHIME® (human fecal inoculum)	↑ Most In proximal, transverse, and distal colon vessels	↑ In proximal and transverse colon vessels	qPCR	*Bifidobacterium* spp. and *Bacteroides-Prevotella* spp. (in proximal colon vessel) *Lactobacillus* spp. (in proximal and transverse colon vessels) *Cl. coccoides-E. rectale* spp. (in proximal and distal colon vessels)	~	Sanchez et al., 2009
AXOS (6-0.26) 10 g day^−1^	3 w	*In vivo* Humans	Nd	Nd	qPCR	*Bifidobacterium* spp. and *Bifidobacterium adolescentis* (in some individuals) in feces	*Lactobacillus* spp. in feces	Cloetens et al., 2010
AXOS (5-0.51) WU-AX (284-0.59) WE-AX (233-0.51) Combinations 5% (m m^−1^)	2 w	*In vivo* Rats	↑ Only for WU-AX, WU-AX + AXOS, and WU-AX + AXOS + WE-AX in cecum and colon	~	qPCR	*Bifidobacterium* spp. (only for AXOS, WE-AX, WE-AX + AXOS, WU-AX + AXOS, WU-AX + AXOS + WE-AX in cecum and WE-AX, WE-AX + AXOS in colon) *Lactobacillus* spp. (only for WU-AX + AXOS in cecum) *Roseburia-E. rectale* spp. (WU-AX, WU-AX + AXOS, WE-AX + AXOS in cecum)	*Lactobacillus* (for AXOS in cecum)	Damen et al., 2011
AX (60-0.70) 10% (m m^−1^)	4 w	*In vivo* Mice	Nd	Nd	qPCR	*Bifidobacterium* spp., *Bacteroides-Prevotella* spp., and *Roseburia* spp. in cecum	~	Neyrinck et al., 2011
AX (60-0.70) 10% (m m^−1^)	6 w	*In vivo* Rats	↑ In cecum and feces	↑ Most In cecum and feces	High-resolution phylogenetic microarray (HITChip)	Eleven bacterial species (e.g., *Bifidobacterium* spp., *Roseburia intestinalis, E. rectale, Collinsella* spp., *Clostridium colinum, Lachnospira pectinoschiza*) in cecum *Akkermansia muciniphila* (in colon)	Nine bacterial species (e.g., *Ruminococcus bromii, Anaerostipes caccae, Eubacterium limosum*, and *A. muciniphila*) in cecum	Van den Abbeele et al., 2011
AXOS (Nd-Nd) 4.8 g day^−1^	3 w	*In vivo* Humans	↓	~	FISH	*Bifidobacterium* spp. in feces	~	Maki et al., 2012
WB (74-0.61) (46-0.63) (42-0.92) (40-0.34) (4-0.22) PSH (300-0.29) (200-0.27) (88-0.16) (72-0.14) 1% (m v^−1^)	48 h	*In vitro* batch fermentation (SHIME® human fecal inoculum)	↑ Especially PSH (300-0.29), (200-0.27), (88-0.16)	↑ Most Especially PSH (200-0.27), (88-0.16), (72-0.14)	Nd	Nd	Nd	Pollet et al., 2012
β-Endoxylanase-treated bread [containing AXOS (18-Nd)] Normal bread [containing AX (174-Nd)] 2.2 g day^−1^	3 w	*In vivo* Humans	↑ In feces	~	FISH	*Bifidobacterium* spp. and *Bacteroides*-*Prevotella* spp. (for treated and normal bread) in feces *Roseburia-E. rectale* spp. and *E. rectale-Cl. coccoides* spp. (only for normal bread) in feces	*Clostridium histolyticum- Clostridium perfringens*	Walton et al., 2012
AX (Nd-Nd) 10 g L^−1^	12 h	*In vitro* batch fermentation (human fecal inoculum)	~	~	Pyrosequencing	*Bacteroides xylanisolvens*	*Blautia* spp.	Yang et al., 2013
AX (Nd-0.55) 17% (m m^−1^)	3 w	*In vivo* Pigs	↑ Most In cecum, proximal colon, transverse colon	↑ In cecum, proximal colon, transverse colon	qPCR	*Bifidobacterium* spp., *Lactobacillus* spp., *F. prausnitzii, R. intestinalis*, and *Blautia coccoides–E. rectale* spp. in feces	~	Nielsen et al., 2014

+ *avDP, average degree of polymerization; A/X, arabinose/xylose ratio; Nd, not determined; ↑, increase of concentration; ↓, decrease of concentration; ~, no significant change; qPCR, quantitative PCR; WU-AX, water-unextractable AX; WE-AX, water-extractable AX; WB, AX and AXOS from wheat bran; PSH, AX and AXOS from Psyllium seed husk*.

##### Bifidogenic effects of AX and AXOS

Several *in vivo* studies (in rodents, pigs, and humans) and *in vitro* studies [during batch and simulator of human intestinal microbial ecosystem (SHIME®) fermentations with fecal slurries] have shown that AX and AXOS are bifidogenic (Table 2). An *in vivo* study with rats has shown that the bifidogenic effect is only caused by AXOS with low average DPs ≤ 5 and A/Xs ≤ 0.27 (Van Craeyveld et al., 2008), whereas other rodent studies have found a stimulation of bifidobacteria by AX and AXOS with high average DPs up to 284 and A/Xs up to 0.70 (Table 2; Damen et al., 2011; Neyrinck et al., 2011; Van den Abbeele et al., 2011). In the latter study, a 60-fold increase of bifidobacteria in the cecum of rats has been found, caused by the consumption of long-chain AX (average DP of 60, A/X of 0.70; Van den Abbeele et al., 2011). Apart from *in vitro* and animal experiments, human studies have revealed a bifidogenic effect caused by a daily intake of 10 g of AXOS per day (Cloetens et al., 2010), 5.5 g of AXOS per day (Maki et al., 2012), and 2.2 g of AX and AXOS per day (Walton et al., 2012; Table 2). However, until recently, many fundamental questions remain unanswered. For instance, how can the low numerical abundant bifidobacteria (<5%) compete with other, more abundant, saccharolytic bacteria in the human colon for AX and AXOS? Do bifidobacteria have a preference for certain AX and AXOS molecules? Are all bifidobacterial strains in the human colon stimulated by AX and AXOS? To answer these questions, a detailed knowledge of the carbohydrate-hydrolyzing capacity of bifidobacteria was missing. Indeed, in the past, studies of the degradation of AX and AXOS through mono-culture fermentations with bifidobacterial strains were restricted to monitoring of bacterial growth, pH, and SCFA production (Van Laere et al., 2000; Crittenden et al., 2002), or fermentations of purified short-chain AXOS standards were performed (Pastell et al., 2009) without revealing the complete fermentation capacity of bifidobacteria. Recently, the mechanistic variations in AXOS degradation by 36 bifidobacterial strains from different donors and origins have been investigated (Rivière et al., 2014). The results show that not all bifidobacterial strains are stimulated by AXOS to the same extent. AXOS degradation by bifidobacteria is complex and involves the consumption of arabinose substituents, whether or not followed by the consumption of the xylose backbones of AXOS, either up to xylotetraose or longer and either intracellularly or extracellularly. Several bifidobacterial strains use the arabinose substituents of AXOS solely, whereas others first consume the arabinose substituents and later import the xylose backbones (up to xylotetraose) into the cell. This extracellular arabinose substituent-oriented metabolism of bifidobacteria has been linked to the presence of genes encoding extracellular cell-associated α-arabinofuranosidases (Lagaert et al., 2010, 2014; Rivière et al., 2014). The majority of the bifidobacterial strains cannot use xylose backbones longer than xylotetraose, i.e., they display a preferential metabolism, except for one strain among the 36 tested ones, *B. catenulatum* LMG 11043^T^, that also uses longer xylose backbones, i.e., they display a non-preferential metabolism (Rivière et al., 2014). This could explain why the bifidogenic effect is strongly influenced by the complexity of the AXOS molecules and decreases with increasing DP (Table 2; Van Craeyveld et al., 2008). A multivariate data analysis of the fermentation data of these 36 bifidobacterial strains has revealed five species-independent clusters, representing five different complementary AXOS degradation mechanisms (Rivière et al., 2014). Cluster I strains, albeit not all, consume free arabinose and xylose; cluster II strains have an extracellular arabinose substituent-oriented metabolism; cluster III strains display a preferential metabolism of non-substituted xylose backbones; cluster IV strains combine the degradation mechanisms of clusters II and III; and cluster V strains display a non-preferential AXOS metabolism. The complementary degradation mechanism of bifidobacterial strains and the ability of intracellular and cell-associated degradation of xylose backbones and AXOS, could explain the selective stimulation of bifidobacteria by AXOS in the presence of other saccharolytic colon bacteria in the human colon. Whole-genome sequence annotations have revealed that some bifidobacterial strains contain genes coding for enzymes involved in the debranching of substituents and in the exo-cleavage of the xylose backbones of AX and AXOS (Schell et al., 2002; van den Broek and Voragen, 2008; van den Broek et al., 2008). Indeed, several AXOS-degrading enzymes have been isolated and characterized in bifidobacterial strains, encompassing β-xylosidases in *B. adolescentis* ATCC 15703 and *B. animalis* subsp. *lactis* BB-12; α-arabinofuranosidases in *B. adolescentis* ATCC 15703, *B. adolescentis* DSM 20083, *B. longum* B667, and *B. longum* NCC2705; and exo-oligoxylanases in *B. adolescentis* LMG 10502 (Lagaert et al., 2010, 2011, 2014). However, up to now, no β-endoxylanases have been found in the genome of bifidobacteria. The only gene (i.e., *BL1543*) that was first annotated as a β-endoxylanase in *B. longum* NCC2705 (Schell et al., 2002) has shown to be an extracellular membrane-associated α-arabinofuranosidase (Lagaert et al., 2010, 2014; Rivière et al., 2014). For the complete utilization of AX, it is likely that most of the *Bifidobacterium* species require cooperation with β-endoxylanase-producing bacteria, such as *Bacteroides* and *Roseburia* species (Chassard et al., 2007; Dodd et al., 2011). For instance, the genome of *R. intestinalis* L1-82 contains three genes possibly encoding β-endoxylanases (NCBI Resource Coordinators, 2014).

##### Butyrogenic effects of AX and AXOS

Besides a bifidogenic effect, AX and AXOS have shown to cause a butyrogenic effect (Table 2). In seven of the 13 *in vitro* and *in vivo* studies summarized in Table 2, bifidobacteria and butyrate-producing colon bacteria (*F. prausnitzii, E. rectale*, and *Roseburia* spp.) are stimulated simultaneously, with a significant increase of butyrate production as a result. As these butyrate-producing colon bacteria are present in high numbers in the colon, a rise in butyrate concentration does not come as a surprise (De Vuyst et al., 2014). In contrast to bifidobacteria, much less is known about the genetic AX- and AXOS-degrading potential of species of butyrate-producing colon bacteria. *In silico* analysis of the genome sequence of, for instance, *E. rectale* ATCC 33656 has shown that there are five genes possibly encoding AXOS-degrading enzymes (exo-oligoxylanase, bifunctional β-xylosidase/α-arabinofuranosidase, β-xylosidase, and two α-arabinofuranosidases; Rivière et al., 2015).

In contrast to ITF, the link between the consumption of AXOS, the bifidogenic effect, and the butyrogenic effect has been assessed only recently (Rivière et al., 2015). It has been shown that a third type of cross-feeding can take place in the presence of AXOS (Figure 4), for instance in the case of *B. longum* NCC2705 (an arabinose substituent degrader of AXOS) and *E. rectale* ATCC 33656 (a complete AXOS degrader). Both strains consume AXOS (as in cross-feeding type 1), but the bifidobacterial strain is additionally stimulated by consuming the monosaccharides released by the extracellular degradation of AXOS by the *E. rectale* strain, leading to cross-feeding interactions that are mutually beneficial (Figure 4). It is likely that these kinds of cross-feeding interactions between bifidobacteria and butyrate-producing colon bacteria, caused by prebiotic consumption, will take place *in vivo* in the human colon (Boets et al., 2013). However, the presence of other bacterial strains, with their own mechanisms of carbohydrate degradation (preferential vs. non-preferential) and own cross-feeding interactions within and between species and genera (Figure 4), complicate the attempts to fully understand the bifidogenic and butyrogenic effects of AX and AXOS in the human colon. Furthermore, the inter-individual variations in bacterial composition make it even more intricate to predict the effects of prebiotic consumption in the colon.

## Conclusions

Human gut microbiota research has grown tremendously over the last years in terms of technology development and implications for human health. For instance, it has been shown that certain key bacteria within the colon, such as bifidobacteria and butyrate-producing colon bacteria, are negatively correlated with disorders such as IBD and colorectal cancer. Of the same importance is the progress that is being made into the modulation of the gut microbiota through the use of probiotics, prebiotics, and FMTs to improve human health. Whereas, in the past, the focus was on straightforward increase of bifidobacterial cell concentrations, shifts in interests are currently emphasizing that the stimulation of butyrate-producing bacteria in the human colon is of importance too. The consumption of prebiotic ITF and AXOS seems to be a promising approach to counteract decreased numbers of bifidobacteria and butyrate-producing colon bacteria. The challenge for the upcoming years will however be to first find out whether these changes in gut microbiota composition are the cause or the consequence of a disorder.

## Author contributions

AR acted as the main author. MS, DL, FL, and LD all contributed substantially to the writing and critical revision of the manuscript and approved its final version.

### Conflict of interest statement

The authors declare that the research was conducted in the absence of any commercial or financial relationships that could be construed as a potential conflict of interest.
